# Myocardial infarction and coronary artery stenosis- An autopsy study

**DOI:** 10.6026/973206300212388

**Published:** 2025-08-31

**Authors:** Anil Kumar Nagar, Noorulla K.A.S, Peeyush Ranjan, Seema Sutay, Shailendra Patel

**Affiliations:** 1 Department of Forensic Medicine and Toxicology, Bundelkhand medical College Sagar, Madhya Pradesh, India; 2 Department of Emergency Medicine, Government Siddhartha Medical College, Vijayawada, Andhra Pradesh, India; 3 Department of Forensic Medicine and Toxicology, Nandkumar Singh Chouhan Government Medical College, Khandwa, Madhya Pradesh, India

**Keywords:** Bundelkhand, cardiovascular diseases, coronary artery stenosis ischemia, myocardial infarction

## Abstract

Autopsy findings related to coronary artery pathology, with particular emphasis on identifying the presence of occlusion by plaques or thrombus and evaluating the degree of coronary artery stenosis is of interest. Hence, out of 110 cases 17 hearts with ischemic changes including 15 males and 2 females were analysed. Location of infarct in LV after staining was in anterior wall of LV, septal wall of LV, papillary muscle of LV and lateral wall was seen in 8, 4, 5 and 2 subjects respectively. There is a significant association between coronary artery stenosis, myocardial ischemia and advancing age, more in males.

## Background:

CVDs (cardiovascular diseases) such as CAD (coronary artery disease) has been reported as leading cause of mortality globally including India posing high burden on the healthcare sector. CAD leads to nearly 25% of all deaths in India making it a substantial health concern. High risk factors prevalence including sedentary lifestyle, obesity, smoking, dyslipidaemia, diabetes and hypertension has raised CAD burden in India. Also, high severity and early onset of CAD in Indians further warrants need for targeted preventive and interventional strategies [1]. In various CAD manifestations, MI (myocardial infarction) is a life-threatening and critical event which results from sudden coronary arteries obstruction from thrombus formation after atherosclerotic plaque rupture. Diagnosis for MI depends on imaging techniques, cardiac biomarkers and electrocardiographic findings, most definitive understanding of true CAD burden is provided by autopsy-based studies. Postmortem assessment allows distinct coronary artery stenosis assessment along with MI, thrombotic occlusion and MI revealing undiagnosed cases [2]. The Global Disease Burden reported that age-standardized CVD death rate in India is 272 per 100,000 population which is significantly higher global mean of 235/100,000 population. This incidence is attributed to significant regional variations in India, a high case fatality rate and premature CAD. In India, 28.1% deaths are from CVD and incidence has sharply increased from 6.9% to 15.2% [3]. CAD prevalence has increased in past few decades owing to increased psychosocial stress, changing dietary patterns and rapid urbanization. Conventional risk factors for CAD including obesity, smoking, dyslipidaemia, diabetes and hypertension does not contribute to its early onset and high prevalence in India. It has been reported that lack of physical activity, low fruit and vegetable consumption, psychological factors, abdominal obesity, diabetes, hypertension, smoking and abnormal lipids act as major contributor leading to 89% population-based MI risk. Indian subjects have higher risk for CAD with comparable risk factors depicting genetic predisposition. Increased lipoprotein a level has been linked to increase in adverse events of dietary changes and urbanization [4]. MI results from prolonged ischemia resulting from coronary arteries occlusion leading to irreversible damage to the myocardium. MI severity is dependent on various factors including duration of ischemia before medical intervention, collateral circulation and location and extent of arterial blockage. Economic CAD burden in India is highly significant. Existing literature data is scarce concerning characteristics and prevalence of myocardial infarction and coronary artery stenosis in various age groups in Indian subjects. Majority of the studies are canter-specific and regional warranting in-depth assessment of various factors causing early CAD onset [5]. Therefore, it is of interest to show the prevalence of coronary artery disease and myocardial infarction severity irrespective of cause of death in the Bundelkhand region (Sagar, Damoh, Chhatarpur, Jhansi and Datia).

## Materials and Methods:

The present prospective duration-based study was aimed to study the prevalence of coronary artery stenosis and severity of myocardial infarction, irrespective of the cause of death, in the Bundelkhand region in subjects that died due to these conditions. The study focused on autopsy findings related to coronary artery pathology, with particular emphasis on identifying the presence of occlusion by plaques or thrombus and evaluating the degree of coronary artery stenosis. The research also aimed to examine the relationship between myocardial ischemia and other related morbid conditions. The study was conducted at the Bundelkhand Medical College and Hospital, located in the Bundelkhand region (Sagar, Damoh, Tikamgarh, Chhatarpur, panna, Jhansi, Datia). The Mortuary of the institution served as the primary location for conducting autopsies and analyzing heart samples. Study Duration the study was carried out over 18 month's duration after approval from Institutional ethical Committee *i.e.*, July 2023 to December 2024. Since the study was based on autopsy findings, no direct interaction with living patients occurred. However, informed consent for post-mortem examinations was obtained from the legal guardians of the deceased, as required by the ethical guidelines for medico-legal investigations. Ethical Approval: The study was approved by the Institutional Ethics Committee of Bundelkhand Medical College and Hospital. All procedures followed ethical guidelines for medical research involving deceased individuals. A total of 110 cases were included in the study. These cases consisted of individuals who had died in the Bundelkhand region and had undergone medicolegal autopsies. The cases included both those who were admitted and died while undergoing treatment, as well as those brought in dead due to coronary artery stenosis or infarction. The inclusion criteria for the study were native cases with coronary artery stenosis or infarction, regardless of the cause of death and cases from the Bundelkhand region were eligible for inclusion. Exclusion Criteria for the study were congenital cardiac malformations (such as Patent Ductus Arteriosus, Atrial Septal Defect and Ventricular Septal Defect), decomposed bodies and bodies with a pacemaker or those that had undergone angioplasty. Severely mutilated bodies (*e.g.*, those with injuries from railway accidents) were also not included.

Concerning post-mortem examination, thoracic cavity was opened following the Virchow's autopsy technique and the heart was removed. The heart was washed thoroughly under running water to remove blood from its chambers. Heart weight was recorded using an electronic weighing machine. For gross examination, the heart was examined for signs of scar tissue indicating old infarctions or coronary artery stenosis. The heart was also checked for areas of softening and hyperemia, as well as any other morbid conditions. Heart weight was recorded using an electronic weighing machine. In coronary artery examination, serial sections of the coronary arteries were made at 3mm intervals to detect any presence of occlusion by plaques or thrombus. The degree of stenosis and the consistency of the coronary arteries. Narrowing of the coronary ostia and the coronary lumen was evaluated for signs of atheromatous plaque or thrombus. In histochemical Staining, two heart slices from randomly selected areas (mid-ventricle to apex) were chosen for histochemical staining to assess areas of myocardial ischemia. TTC Solution was prepared with 12 grams of sodium Dihydrogen phosphate is dissolved in one litre of distilled water to make 0.1 M solution. 14.2 grams of Disodium hydrogen phosphate is dissolved in one litre of distilled water to make 0.1 M solution. Ph of the solution was adjusted by mixing 0.1 M disodium hydrogen phosphate solution and 0.1 M sodium dihydrogen phosphate in different proportion as follows: of Triphenyl Tetrazolium Chloride was then dissolved in one litre of above phosphate buffer solution of pH 7.8 to make 1% TTC solution. 1% TTC solution was stored in Amber coloured bottle as the dye TTC is photosensitive, gets inactivated on exposure to light.

For staining, heart slices were washed with running water and wiped with tissue paper and placed in a plastic container of size larger than the heart slice. One container was used for each slice. TTC solution was poured into the container containing heart slice, so that the solution level in the container was about 2 cm above the heart slice to prevent atmospheric oxygen penetration. Then the system in placed in a cardboard board box to avoid light exposure. The incubation is carried out at room temperature for 20 minutes. Ten minutes after incubation, the heart slice was turned upside down to prevent artefactual nonstaining. After incubation for 20 minutes, heart slices were removed and examined for unstained areas of myocardium. Normal myocardium-stained brick red whereas infarct area remained unstained or shows very much reduced staining. The positive result was inferred by suspected infarcted detection of grossly inapparent Mis with TTC is a useful adjunct in the diagnosis of acute myocardial ischemia. The data obtained from the examination of coronary arteries and heart tissue was subjected to descriptive statistical analysis. The prevalence of coronary artery stenosis and infarction was analysed. The degree of stenosis and occlusion in coronary arteries was evaluated and categorized. Histopathological findings from the staining of heart slices were analysed for correlation with coronary artery pathology. The study findings were represented as frequencies and percentages and appropriate statistical tests (*e.g.*, chi-square tests) were applied to identify significant differences or associations. Data analysis was performed using Microsoft Excel and SPSS 25.0 version software to ensure accuracy and reliability of the findings. For qualitative data, percentages were calculated to represent the distribution of categorical variables. For quantitative data, the mean and standard deviation were determined to summarize continuous variables, including the extent of coronary artery stenosis and infarct severity.

## Results:

The study comprised of 110 cases that were brought to mortuary of Bundelkhand region for medicolegal autopsy. Cases, showing signs of decomposition were not considered. Cases studied included deaths due to Road traffic accidents, burns, poisoning, asphyxia and cases presenting as sudden deaths. Cases presenting as sudden unexpected deaths with history suggestive of heart disease were especially included. Detailed histories as to be circumstances leading to death, any past history of myocardial infarction, or symptoms suggestive of heart disease like, breathlessness, chest pain, collapse, were obtained from the relatives, inquest papers and wherever possible from the hospital records. In none of the cases was diagnosis of myocardial infarction made. In all the cases that presented as sudden death either the patient was found dead or was declared in the hospital as "brought dead". Majority of the study subjects were in the range of 21-30 years with 26 males and 6 females followed by 26 subjects from 31-40 years with 24 males and 2 females, 13 in each 41-50 and 51-60 years, 11 in 11-20 years, 8 in 61-70 years, 3 in 0-10 years, 2 in 71-80 years and no subject in 81-90 years among total 110 subjects. There were 2.73% (n=90) males and 10% (n=20) females in the study. The most common cause of death in study subjects was head injury in 41.8% (n=46) subjects followed by burns in 24.5% (n=27), sudden unexpected death in 18.18% (n=20), hemorrhagic shock and pulmonary tuberculosis in 8.18% (n=9), suspected MI in 5.45% (n=6), suspected poisoning and history was not available in 4.55% (n=5) subjects each and asphyxia in 2.73% (n=3) subjects (Table 1 - see PDF). On assessing the incidence of stenosis of the various arteries in study subjects, R1 had grade 0, 1, 2, 3 and 4 in 24, 53, 25, 7 and 1 subject among total 782% (n=86) subjects. R2 had grade 0, 1, 2, 3 and 4 in 46, 44, 10, 1 and 4 subjects among total 53.6% (n=59) subjects. R3 was seen in had grade 0, 1, 2, 3 and 4 in 75, 23, 4, 2 and 0 subject among total 26.3% (n=29) subjects. L1 had grade 0, 1, 2, 3 and 4 in 24, 53, 25, 7 and 0 subject among total 77.3% (n=85) subjects. L2 had grade 0, 1, 2, 3 and 4 in 24, 45, 22, 14 and 5 subjects among total 78.2% (n=86) subjects. L3 had grade 0, 1, 2, 3 and 4 in 47, 38, 12, 4 and 3 subjects among total 52.7% (n=58) subjects (Table 2 - see PDF). Concerning overview of the demographics and methodology used to examine the hearts for ischemic changes, there were 17 hearts with ischemic changes including 15 males and 2 females. There were 6 subjects aged <45 years and 11 subjects >45 years. There were 11 obese subjects and staining method used was NBT and TTC (Table 3 - see PDF). Survival period in study subjects was 0-4 days, 4-8 days, 8-12 days and unknown in 4, 5, 3 and 6 study subjects respectively (Table 4 - see PDF). For infarct type and its location in LV after staining, complicating, transmural and subendocardial type were seen 3, 6 and 8 subjects. In subendocardial, localized and total type was seen in 4 subjects each. Location of infarct in LV after staining was in anterior wall of LV, septal wall of LV, papillary muscle of LV and lateral wall was seen in 8, 4, 5 and 2 subjects respectively (Table 5 - see PDF).

## Discussion:

Atherosclerosis was the predominant lesion observed in all coronary arteries in this study. Findings show that coronary atheromatous changes are nearly universal in adults over 30, with the youngest male and female showing lesions at 22 and 25 years, respectively. Thus, it is more appropriate to categorize cases based on the severity of stenosis rather than the presence or absence of atherosclerosis. These results align with Kumar *et al.* in 2020 [6]. Previous studies supported our findings including studies by Rao *et al.* in 2014 [7] where authors found CAD in 56.86% of 204 sudden cardiac deaths in Bangalore, with the LAD most commonly affected, especially in males aged 50-60. Rao *et al.* in 2014 observed coronary stenosis in 34% of males and 20% of females, with 23.6% of individuals under 40 showing significant narrowing, again with LAD predominance [7]. This study was similar to the study by Bansal *et al.* 2015 [8] where authors using the Modified AHA Classification, found most coronary lesions in the LAD, predominantly Grade 3 and 4. Our grading-based analysis also showed a strong correlation between stenosis severity and ischemic changes, with LAD being the most frequently affected artery. Thrombosis without atherosclerosis was not seen in our study, contradicting Bagnall *et al.* in 2016 [9] who argued that thrombosis only occurs over mural disease. Our study found a greater prevalence and severity of lesions in males. Although Gastanadui *et al.* in 2024 [10] questioned sex-based differences; our data aligns with the broader literature in showing male predominance. Patients with myocardial ischemia had significantly higher mean age, though BMI and sex differences were not statistically significant. Notably, individuals over 50 were 13 times more likely to show ischemic changes than those under 50. In the present study, subendocardial infarction of the right ventricle was observed in 1 case (6%), consistent with Thej *et al.* in 2012 [11] who reported a similar incidence. All other subendocardial infarcts involved the left ventricle. Subendocardial infarcts were noted in approximately 55% of cases (11 total), consistent with Thej *et al.* in 2012 [11]. These mostly involved elderly patients with advanced atherosclerosis, often in shock or terminal illness. Coronary thrombosis was confirmed in only onetrans mural infarct, supporting Bhanvadia *et al.* 2013 [12] who observed low thrombosis incidence in sub endocardial infarcts. This study examined 110 hearts; 4 weighed >450g (3 of them 500g). Of these, 2 showed no infarcts despite low-grade stenosis-possibly due to unknown survival duration. The other 2 had infarcts (one transmural with mitral incompetence, the other total sub endocardial infarct), showing that hypertrophied hearts can sustain both infarct types. The few limitations of the present study include caution in generalizing results, incomplete data on survival time and missing information on other factors.

## Conclusion:

The presence of ischemic changes correlates with higher mean stenosis severity and older age groups. Fresh myocardial infarctions are more reliably detected using histochemical staining methods than with gross macroscopic examination. Histochemical techniques offer added diagnostic value in detecting myocardial ischemia, particularly when conventional methods may not reveal early or subtle changes. Future research should focus on expanding the sample size to include living subjects to better understand the progression of coronary artery disease and ischemia in different populations.

## References

[1] Menon J.C (2024). Trials..

[2] Kalra A (2023). Lancet Reg Health Southeast Asia..

[3] Loitongbam L, Surin W.R. (2024). Cureus..

[4] Sasikumar M (2023). Indian Heart J..

[5] Ardeshna D.R (2018). Ann Transl Med..

[6] Kumar SA (2020). Medical Journal Armed Forces India..

[7] Rao D (2014). Emerg (Tehran)..

[8] Bansal Y.S (2015). J Clin Diagn Res..

[9] Bagnall R.D (2016). Engl J Med..

[10] Gastanadui M.G (2024). Arterioscler Thromb Vasc Biol..

[11] Thej M.J (2012). J Cardiovasc Dis Res..

[12] Bhanvadia V.M (2013). J Clin Diagn Res..

